# Case Report: Immune-related eruptive keratoacanthoma with fungal coinfection under PD-1 inhibitor therapy: a diagnostic and therapeutic challenge

**DOI:** 10.3389/fphar.2025.1619450

**Published:** 2025-08-13

**Authors:** Ying-Jie Su, Zheng Wu, Yan-Li Hou, Meng-Xia Yan, Xiu-Mei Ma, Hou-Wen Lin

**Affiliations:** ^1^ Department of Pharmacy, Shanghai Jiao Tong University School of Medicine Affiliated Renji Hospital, Shanghai, China; ^2^ Department of Radiation Oncology, Shanghai Jiao Tong University School of Medicine Affiliated Renji Hospital, Shanghai, China; ^3^ Department of Pharmacy, Ningbo Hangzhou Bay Hospital, Ningbo, Zhejiang, China

**Keywords:** immune checkpoint inhibitors, programmed cell death protein 1, immune-related adverse event, immune-related cutaneous adverse event, eruptive keratoacanthomas

## Abstract

**Background:**

It has been reported that immunotherapy with programmed cell death protein 1 (PD-1) inhibitors (pembrolizumab or nivolumab) can induce multiple eruptive keratoacanthomas (KAs), representing an immune-related cutaneous adverse event (ircAE).

**Methods:**

This case report describes a 63-year-old female with recurrent cervical adenocarcinoma who developed multiple eruptive KAs and a concurrent fungal infection following treatment with the PD-1 inhibitor zimberelimab. We analyzed the etiology, diagnosis, and treatment by integrating clinical manifestations, pathological examinations, previous treatment history, and a review of the literature.

**Results:**

Despite an initial misdiagnosis as a fungal infection, multidisciplinary review identified KA as an ircAE. Topical corticosteroids led to resolution, and another PD-1 inhibitor was reintroduced without recurrence of cutaneous toxicity.

**Conclusion:**

This is the first documented case of eruptive KA linked to zimberelimab, expanding the spectrum of PD-1 inhibitor-associated ircAEs. A concurrent fungal infection obscured the diagnosis, delaying appropriate treatment and highlighting the importance of recognizing rare ircAEs and multidisciplinary collaboration.

## 1 Introduction

Adverse events (AEs) associated with immune checkpoint inhibitor (ICI) therapy are termed immune-related adverse events (irAEs), arising from dysregulated immune activation. Among these, immune-related cutaneous adverse events (ircAEs) represent the most frequently observed toxicities, with epidemiologic studies indicating an incidence exceeding 50% across all grades (Haanen et al., 2022; Schneider et al., 2021). The clinical spectrum of ircAEs demonstrates significant variability, predominantly characterized by non-specific maculopapular eruptions, inflammatory dermatitis, or vitiligo (Haanen et al., 2022; Schneider et al., 2021). Keratoacanthoma (KA) is common and somewhat cryptic tumor in humans, with controversial epidemiology, histopathological diagnostic criteria, prognosis, and treatment guidelines (Kwiek and Schwartz, 2016). It has been reported that immunotherapy with programmed cell death protein 1 (PD-1) inhibitors (pembrolizumab or nivolumab) can induce multiple eruptive KAs, representing a rare ircAE (Freites-Martinez et al., 2017; Crow et al., 2020; Bandino et al., 2017). We present the particularly unique concurrence of multiple eruptive KAs with a fungal infection in a cervical adenocarcinoma patient receiving another PD-1 inhibitor (zimberelimab), emphasizing diagnostic challenges and management.

## 2 Case presentation

A 63-year-old female diagnosed with second recurrence cervical adenocarcinoma was admitted to a tertiary teaching hospital in Shanghai and was treated with chemotherapy and zimberelimab immunotherapy for second-line therapy from 7 February 2024, achieving a partial response (PR) after four cycles. Maintenance therapy with zimberelimab was subsequently initiated after return to the local hospital.

**FIGURE 1:**
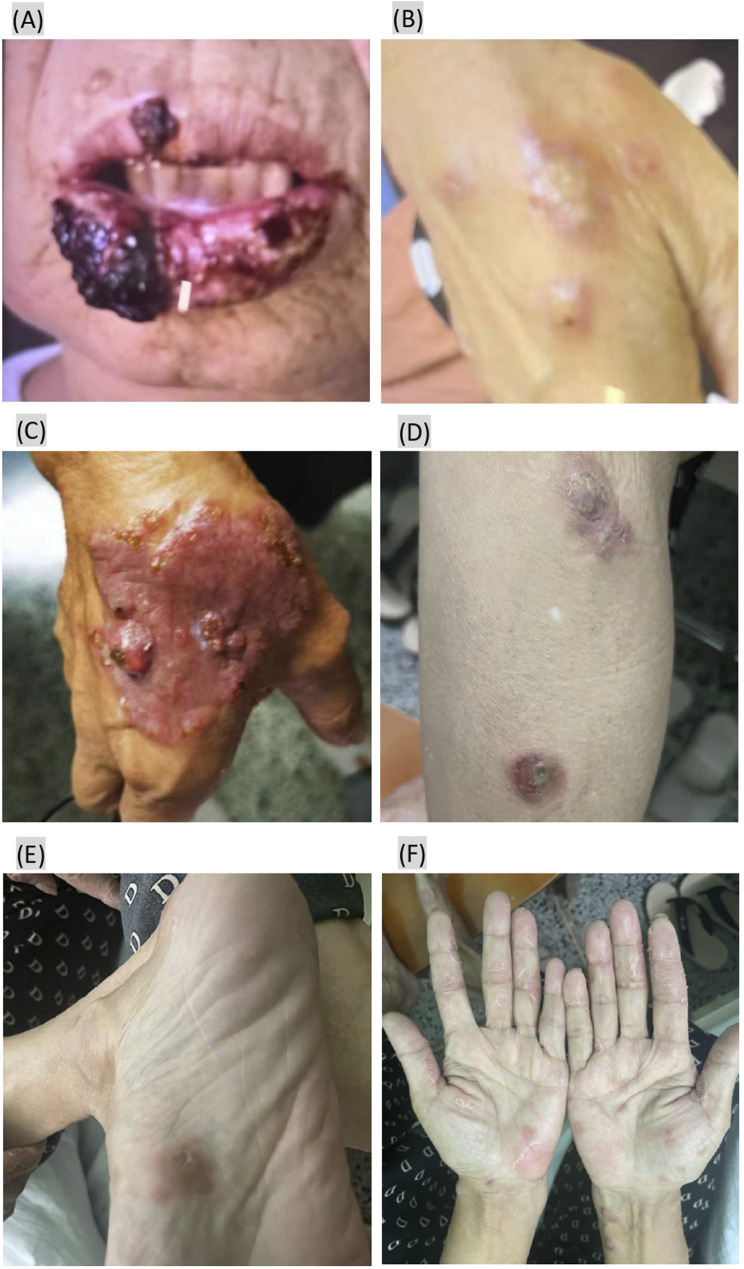
Eruptive keratoacanthoma papules presenting after starting zimberelimab treatment. **(A,B)** Papules on the lips and right dorsal hand 5 months after zimberelimab treatment. **(C)** The lesions on right dorsal hand had worsened 6 months after zimberelimab treatment. **(D–F)** New papules appeared on her lower legs, soles of the feet, and palms nearly 7 months after zimberelimab treatment.

The patient presented on May 20 with scattered pruritic nodules measuring approximately 2–5 mm, surrounded by erythema, on her lips, oral mucosa, and extremities. By July, the lip lesions had progressed to ulceration and scab formation (Figure 1A), and new multiple pruritic papules appeared on the dorsal aspect of the right hand (Figure 1B). By August 8, the lesions on the right dorsal hand had worsened, and a large violaceous nodule with hard papules and areas of ulceration was observed (Figure 1C). A biopsy of the lesion from the right dorsal hand performed at a provincial dermatology hospital led to a diagnosis of superficial fungal infection, and she was prescribed antifungal medicines. However, by late August, new papules developed on her lower legs, soles of the feet, and palms during antifungal treatment (Figures 1D–F). She was switched to other antifungal medicines, but there was still no improvement.

## 3 Diagnosis and treatment

**FIGURE 2:**
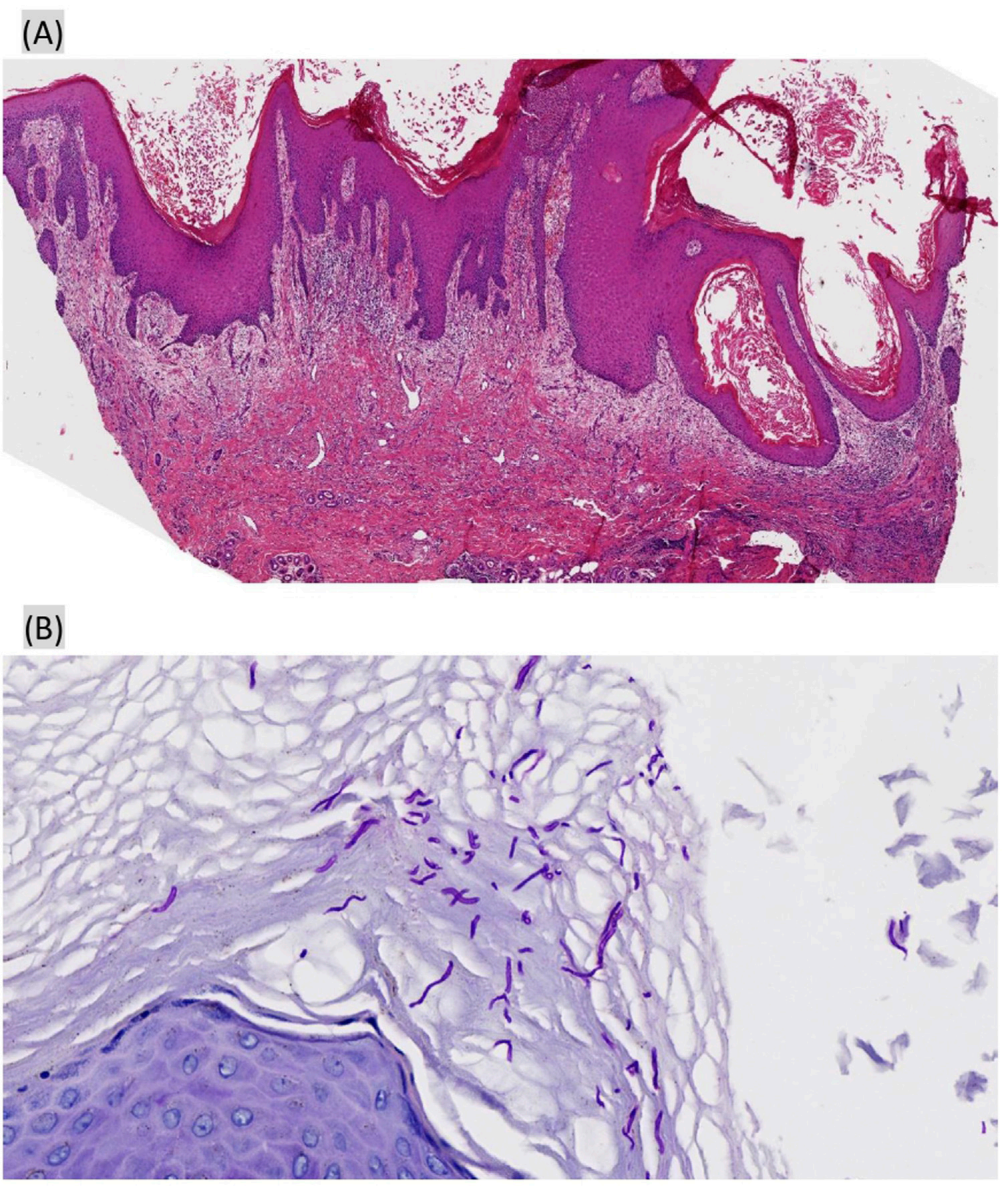
Biopsy of the right dorsal hand papules. **(A)** Hematoxylin–eosin (HE), original magnification ×40: excessive keratosis, epidermal hyperplasia and hypertrophy, acanthosis, and pseudoepitheliomatous hyperplasia; **(B)** periodic acid-Schiff (PAS) ×400: fungal spores and mycelia were found in the corneum and epidermal granular layers.

**FIGURE 3:**
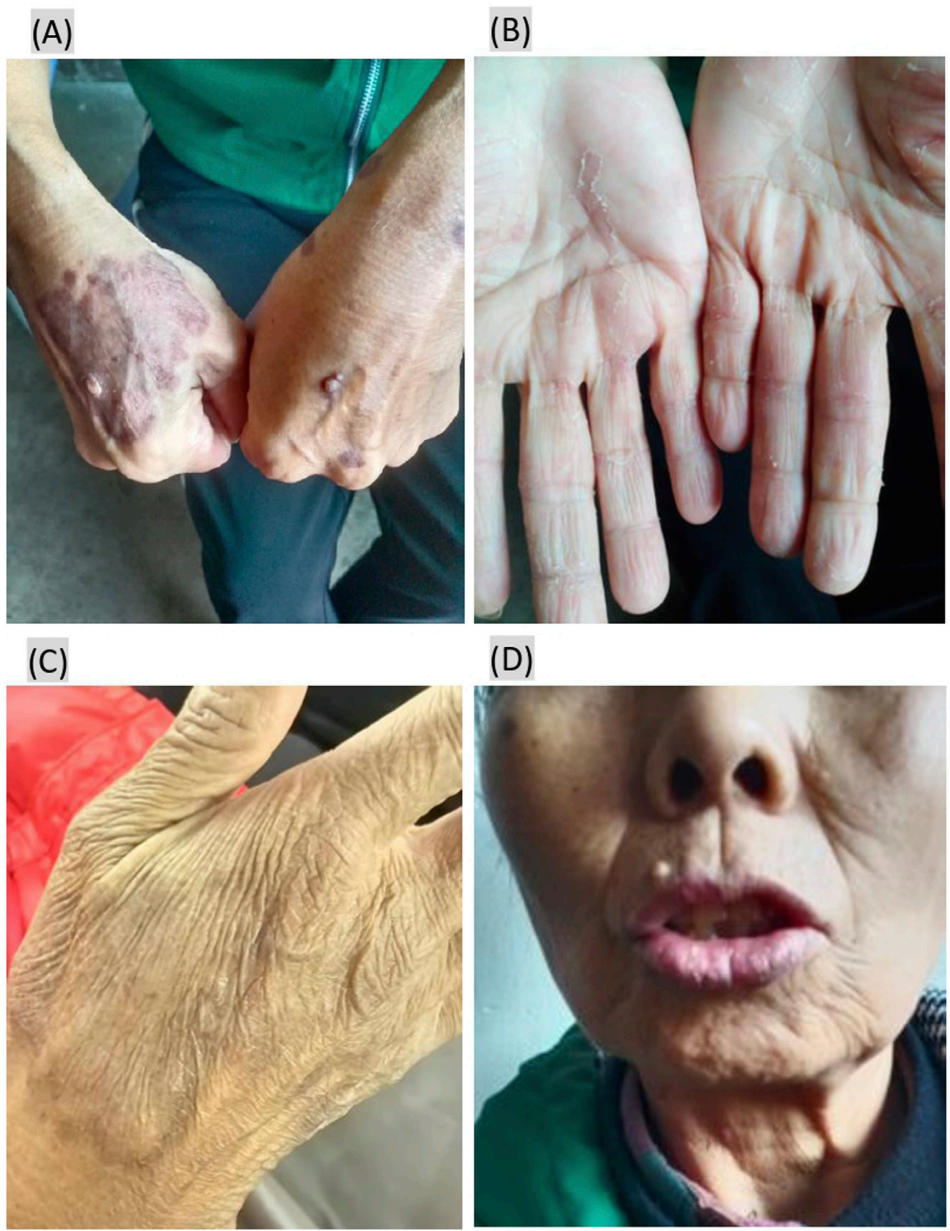
The skin lesions improved after treatment with a topical halomethasone cream. **(A,B)** All papules improved 1 month after treatment with a topical halomethasone cream; **(C,D)** skin lesions had completely regressed after 5 months after topical halomethasone treatment.

In September, the patient returned to the tertiary teaching hospital in Shanghai for further treatment, where the original antifungal therapy was continued following a dermatology consultation. An oncology clinical pharmacist conducted a detailed review of the patient’s pathological report in detail: 1. Microscopic pathological findings (Figure 2A) showed excessive keratosis, epidermal hyperplasia and hypertrophy, acanthosis, and irregular extension of dermal processes, along with pseudoepitheliomatous hyperplasia. A neutrophilic abscess, edema of the dermal papillae, small vessel hyperplasia, inflammatory cell infiltration—primarily lamellar lymphocytes and neutrophils—were observed in the epidermis; no granulomas were identified. 2. PAS staining (Figure 2B) presented fungal spores, and mycelia were found in the corneum and epidermal granular layers. Except for fungal infection, the pathological findings were similar to the appearance of “multiple eruptive KAs,” a rare ircAE by ICIs reported in previous papers (Schwartz et al., 2022; Bandino et al., 2017; Freites-Martinez et al., 2017; Crow et al., 2020; Feldstein et al., 2018). The association between the eruptive KAs and zimberelimab was determined using the Naranjo scores, with scores of 5 implying a “probable” relationship. The pharmacist recommended topical halomethasone cream (twice daily) alongside antifungal agents, and zimberelimab was discontinued due to stable tumor status. Most of the skin lesions improved after 10 days of topical halomethasone treatment, and all showed significant remission after 1 month (Figures 3A,B). No new skin lesions were found. Halomethasone cream was subsequently discontinued, and only antifungal therapy was maintained. Another PD-1 inhibitor (sintilimab) was re-administered in the local hospital from December 2024. As of 10 February 2025, the patient had received five infusions of sintilimab, during which time her primary disease remained stable and the skin lesions had completely regressed (Figures 3C,D).

## 4 Discussion

To our knowledge, this is the first published report of eruptive KA after treatment with zimberelimab, a PD-1 inhibitor, occurring concurrently with a skin fungal infection. This case expands the spectrum of PD-1 inhibitor-associated ircAEs. While it has been reported that PD-1 inhibitors (pembrolizumab or nivolumab) can induce multiple eruptive KAs, this is the first such association documented with zimberelimab.

Eruptive KA is a rare ircAE induced by immunotherapy, with pathological manifestations ranging from atypical pseudoepitheliomatous hyperplasia (Lee and Wang, 2020; Chaudhari et al., 2017) to typical keratoacanthomas (Schwartz et al., 2022; Olsen et al., 2024; Bednarek et al., 2018) and, in some cases, even resembling squamous cell carcinoma (SCC) (Marsh et al., 2020; Antonov et al., 2019). According to the data from the FAERS database, the current incidence rates of KA and cutaneous squamous cell carcinoma (cSCC) caused by ICIs are 0.027% (43/158000) and 0.053% (83/158000), respectively (Aggarwal et al., 2024). Most cases of eruptive KAs in patients receiving immunotherapy have been reported in the context of lichenoid dermatitis (Schwartz et al., 2022; Feldstein et al., 2018), and eruptive KAs have also been associated with bullous eruptions (Bandino et al., 2017; Schwartz et al., 2022), vitiliginous patches (Bandino et al., 2017), and spongiotic dermatitis (Crow et al., 2020). The patient described in this study exhibited atypical hyperkeratosis, pseudoepitheliomatous hyperplasia, and fungal infection in the context of extensive inflammatory cell infiltration, making the ircAE more complex and difficult to differentiate. This led to the use of antifungal therapy alone during the initial months, delaying appropriate treatment for eruptive KA. As a result, the patient’s quality of life reduced and treatment costs increased. To date, we were the only group to have identified a case of ICI-induced KA occurring concurrently with a skin infection, which contributed to the delayed diagnosis. This case was of great significance in reminding oncologists and dermatologists to highlight a differential diagnosis for rare ircAEs, reinforcing the need for simultaneous biopsy and culture in new lesions. Similarly, there have been analogous reports of such patients being misdiagnosed with SCC and receiving unnecessary radiotherapy, chemotherapy, and/or targeted therapy (Crow et al., 2020; Lee and Wang, 2020). In view of this, rare irAEs particularly require a detailed differential diagnosis that takes into account both pathological methods (Wang and Chen, 2025) and the entire course of diagnosis and treatment.

Multiple eruptive KAs pose a great treatment challenge due to the large number of lesions and the morbidity associated with both surgical and medical management. As a result, controversies remain regarding the optimal approach. In some fortunate patients, the lesions resolved spontaneously, even when immunotherapy was not discontinued (Haraszti et al., 2019) or after the largest lesion was treated with complete curettage, with other lesions resolving thereafter (Antonov et al., 2019). Some patients, like the one we reported in this study, achieved complete regression with only the topical application of a potent corticosteroid cream (Fradet et al., 2019; Marsh et al., 2020); others received combinations of oral corticosteroids (Bandino et al., 2017), acitretin (Schwartz et al., 2022; Haraszti et al., 2019) or hydroxychloroquine (Crow et al., 2020), or intralesional injections of triamcinolone (Lee and Wang, 2020; Freites-Martinez et al., 2017; Bednarek et al., 2018) or 5-fluorouracil (Olsen et al., 2024). In addition, surgical resection or cryosurgery has been used to treat KA lesions in some cases (Marsh et al., 2020; Freites-Martinez et al., 2017; Feldstein et al., 2018; Chaudhari et al., 2017). According to previous literature and this case, eruptive KAs represent inflammation-related, reactive, reversible hyperplasia, rather than true neoplasia, supporting the use of nonsurgical anti-inflammatory therapies (Crow et al., 2020).

## 5 Conclusion

This case underscores the need for heightened suspicion of ircAEs, like eruptive KAs in patients on ICIs, even in the presence of concurrent infections. Multidisciplinary teamwork (MDT) facilitates timely diagnosis, and topical corticosteroids offer a non-invasive treatment option. Successful rechallenge with a different PD-1 inhibitor suggests appropriate treatment and careful monitoring may enable continued immunotherapy.

## Data Availability

The original contributions presented in the study are included in the article/supplementary material; further inquiries can be directed to the corresponding author.

## References

[B1] Aggarwal P. Clark D. Shah A. Rismiller K. Neltner S. A. (2024). Keratoacanthoma and cutaneous squamous cell carcinoma with PD-1 and PD-L1 inhibitor use. JAMA Dermatol 160, 573–575. 10.1001/jamadermatol.2024.0390 38598190 PMC11007655

[B2] Antonov N. K. Nair K. G. Halasz C. L. (2019). Transient eruptive keratoacanthomas associated with nivolumab. JAAD Case Rep. 5, 342–345. 10.1016/j.jdcr.2019.01.025 31049378 PMC6476889

[B3] Bandino J. P. Perry D. M. Clarke C. E. Marchell R. M. Elston D. M. (2017). Two cases of anti-programmed cell death 1-associated bullous pemphigoid-like disease and eruptive keratoacanthomas featuring combined histopathology. J. Eur. Acad. Dermatol Venereol. 31, e378–e380. 10.1111/jdv.14179 28222231

[B4] Bednarek R. Marks K. Lin G. (2018). Eruptive keratoacanthomas secondary to nivolumab immunotherapy. Int. J. Dermatol 57, e28–e29. 10.1111/ijd.13893 29318617

[B5] Chaudhari S. Leon A. Levin E. Neuhaus I. Liao W. (2017). Case report of multiple keratoacanthomas and squamous cell carcinomas in a patient receiving pembrolizumab. J. Drugs Dermatol 16, 513–515. 28628690

[B6] Crow L. D. Perkins I. Twigg A. R. Fassett M. S. Leboit P. E. Berger T. G. (2020). Treatment of PD-1/PD-L1 inhibitor-induced dermatitis resolves concomitant eruptive keratoacanthomas. JAMA Dermatol 156, 598–600. 10.1001/jamadermatol.2020.0176 32211828

[B7] Feldstein S. I. Patel F. Larsen L. Kim E. Hwang S. Fung M. A. (2018). Eruptive keratoacanthomas arising in the setting of lichenoid toxicity after programmed cell death 1 inhibition with nivolumab. J. Eur. Acad. Dermatol Venereol. 32, e58–e59. 10.1111/jdv.14503 28776778

[B8] Fradet M. Sibaud V. Tournier E. Lamant L. Boulinguez S. Brun A. (2019). Multiple keratoacanthoma-like lesions in a patient treated with pembrolizumab. Acta Derm. Venereol. 99, 1301–1302. 10.2340/00015555-3301 31449315

[B9] Freites-Martinez A. Kwong B. Y. Rieger K. E. Coit D. G. Colevas A. D. Lacouture M. E. (2017). Eruptive keratoacanthomas associated with pembrolizumab therapy. JAMA Dermatol 153, 694–697. 10.1001/jamadermatol.2017.0989 28467522 PMC5523926

[B10] Haanen J. Obeid M. Spain L. Carbonnel F. Wang Y. Robert C. (2022). Management of toxicities from immunotherapy: ESMO Clinical Practice Guideline for diagnosis, treatment and follow-up. Ann. Oncol. 33, 1217–1238. 10.1016/j.annonc.2022.10.001 36270461

[B11] Haraszti S. Polly S. Ezaldein H. H. Rothbaum R. Delost G. R. Beveridge M. (2019). Eruptive squamous cell carcinomas in metastatic melanoma: an unintended consequence of immunotherapy. JAAD Case Rep. 5, 514–517. 10.1016/j.jdcr.2019.03.014 31205993 PMC6558268

[B12] Kwiek B. Schwartz R. A. (2016). Keratoacanthoma (KA): an update and review. J. Am. Acad. Dermatol 74, 1220–1233. 10.1016/j.jaad.2015.11.033 26853179

[B13] Lee M. P. Wang A. R. Wang A. R. (2020). Pembrolizumab-induced pseudoepitheliomatous eruption consistent with hypertrophic lichen planus. J. Cutan. Pathol. 47, 275–279. 10.1111/cup.13587 31589773

[B14] Marsh R. L. Kolodney J. A. Iyengar S. Yousaf A. Louden B. A. Al-Bouri A. (2020). Formation of eruptive cutaneous squamous cell carcinomas after programmed cell death protein-1 blockade. JAAD Case Rep. 6, 390–393. 10.1016/j.jdcr.2020.02.024 32382626 PMC7200186

[B15] Olsen E. Svoboda S. A. Montanez-Wiscovich M. Saikaly S. K. (2024). Multiple eruptive keratoacanthomas secondary to nivolumab immunotherapy. J. Immunother. 47, 98–100. 10.1097/cji.0000000000000498 38009069

[B16] Schneider B. J. Naidoo J. Santomasso B. D. Lacchetti C. Adkins S. Anadkat M. (2021). Management of immune-related adverse events in patients treated with immune checkpoint inhibitor therapy: ASCO guideline update. J. Clin. Oncol. 39, 4073–4126. 10.1200/jco.21.01440 34724392

[B17] Schwartz R. J. Ho G. Smith A. Collgros H. Regio Pereira A. Gouveia B. (2022). Successful treatment of eruptive keratoacanthomas with actitretin for patients on checkpoint inhibitor immunotherapy. J. Eur. Acad. Dermatol Venereol. 36, e445–e448. 10.1111/jdv.17940 35043483

[B18] Wang L.-H. Chen C.-C. (2025). Extensive bullous lichenoid drug eruption associated with the combination therapy of enfortumab vedotin and pembrolizumab: a case report. Dermatol. Sin. 43, 67–68. 10.4103/ds.ds-d-23-00188

